# CRF-R1 activation in the anterior-dorsal BNST induces maternal neglect in lactating rats via an HPA axis-independent central mechanism

**DOI:** 10.1016/j.psyneuen.2015.11.015

**Published:** 2016-02

**Authors:** Stefanie M. Klampfl, Paula J. Brunton, Doris S. Bayerl, Oliver J. Bosch

**Affiliations:** a Department of Behavioural and Molecular Neurobiology, University of Regensburg, 93053 Regensburg, Germany; b Division of Neurobiology, The Roslin Institute and R(D)SVS, University of Edinburgh, Midlothian EH25 9RG, UK

**Keywords:** Corticotropin-releasing factor, Bed nucleus of the stria terminalis, Maternal behavior, Anxiety, Hypothalamic–pituitary–adrenal axis

## Abstract

Adequate maternal behavior in rats requires minimal corticotropin-releasing factor receptor (CRF-R) activation in the medial-posterior bed nucleus of the stria terminalis (mpBNST). Based on the architectural heterogeneity of the BNST and its distinct inter-neural connectivity, we tested whether CRF-R manipulation in another functional part, the anterior-dorsal BNST (adBNST), differentially modulates maternal behavior.

We demonstrate that in the adBNST, activation of CRF-R1 reduced arched back nursing (ABN) and nursing, whereas activation of CRF-R2 resulted in an initial reduction in nursing but significantly increased the incidence of ABN 5 h after the treatment. Following stressor exposure, which is detrimental to maternal care, ABN tended to be protected by CRF-R1 blockade. Maternal motivation, maternal aggression, and anxiety were unaffected by any manipulation. Furthermore, under basal and stress conditions, activation of adBNST CRF-R1 increased plasma ACTH and corticosterone concentrations, whereas stimulation of adBNST CRF-R2 increased basal plasma ACTH and corticosterone concentrations, but blocked the stress-induced increase in plasma corticosterone secretion. Moreover, both the CRF-R1 and -R2 antagonists prevented the stress-induced increase in plasma corticosterone secretion. Importantly, elevated levels of circulating corticosterone induced by intra-adBNST administration of CRF-R1 or -R2 agonist did not impact maternal care. Finally, *Crf* mRNA expression in the adBNST was increased during lactation; however, *Crfr1* mRNA expression was similar between lactating and virgin rats.

In conclusion, maternal care is impaired by adBNST CRF-R1 activation, and this appears to be the result of a central action, rather than an effect of elevated circulating levels of CORT. These data provide new insights into potential causes of disturbed maternal behavior postpartum.

## Highlights

• CRF-R1 activation in the adBNST reduces arched back nursing and nursing.
• adBNST CRF-R2 activation increases arched back nursing but impairs overall nursing.
• Inhibiting adBNST CRF-R1 prevents the stress-induced decline in arched back nursing.
• CRF-R activation in the adBNST stimulates the HPA axis in a stress-dependent manner.
• CRF-R-induced HPA axis activation is not responsible for reduced maternal care.

## 1 Introduction

The peripartum period is accompanied by numerous physiological and behavioral adaptations organized by the maternal brain. These changes are essential for the adequate expression of maternal behavior, thereby ensuring the proper development of the offspring, as well as for the mothers’ mental health. Indeed, up to 30% of mothers that develop postpartum mood disorders show child neglect, with some committing infanticide (Brummelte and Galea, 2010b, Friedman and Resnick, 2009).

One peptidergic system that evidently contributes to such maladaptations during this highly sensitive period is the central corticotropin-releasing factor (CRF) system (Klampfl et al., 2014, Klampfl et al., 2013, Magiakou et al., 1996). The CRF system consists of CRF and its related peptides, urocortin 1, 2 and 3, which bind to CRF receptor type 1 (CRF-R1), CRF-R2, and the CRF binding protein with different affinities (Reul and Holsboer, 2002). CRF was first discovered as the main initiator of the hypothalamic–pituitary–adrenal (HPA) axis and is the major secretagogue of ACTH from the anterior pituitary into the portal blood system (Vale et al., 1981). ACTH stimulates the release of corticosterone (CORT) from the adrenal glands which plays several roles in mediating appropriate responses to stress and also exerts negative feedback control of the HPA axis at the level of the hippocampus, paraventricular nucleus (PVN), and pituitary. Moreover, CRF and its related peptides also exert central functions and influence a variety of non-social and social behaviors, e.g., increased anxiety-related behavior (Britton et al., 1986, Klampfl et al., 2014, Klampfl et al., 2013, Sahuque et al., 2006) and reduced maternal behavior (Gammie et al., 2004, Klampfl et al., 2014, Klampfl et al., 2013, Pedersen et al., 1991), even leading to pup killing in virgin rats (Pedersen et al., 1991). Intriguingly, some of these effects can be attributed to the CRF system of the bed nucleus of the stria terminalis (BNST) (Klampfl et al., 2014, Sahuque et al., 2006).

The BNST is a complex and particularly heterogeneous structure within the limbic system. It acts as a central relay site for the integration of a variety of neuronal signals, mediating behavioral as well as physiological responses. The BNST can be roughly divided into anterior and posterior divisions. The anterior part is mainly connected with hypothalamic and brainstem regions associated with autonomic activity (Dong et al., 2001a), while the posterior division is involved in controlling neuroendocrine systems and social behaviors (Dong et al., 2001a, Dong and Swanson, 2004). Such heterogeneity within a single brain region raises the question whether social (e.g., maternal behavior) and non-social behaviors (e.g., anxiety) might be differentially regulated within the BNST depending on the specific subdivision.

**Fig. 1:**
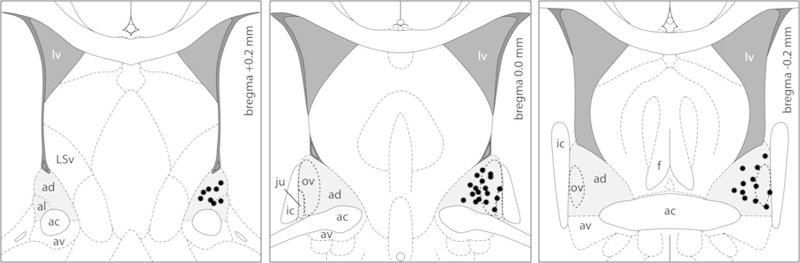
Histological localization of infusion cannula within the adBNST. Histological definition of the adBNST containing a variety of subnuclei according to Dong et al. (2001b) shown on schematic plates adapted from the stereotaxic rat brain atlas (Paxinos and Watson, 1998). For better visualization purposes, correct cannula placement sites (black dots) for subsequent drug infusion in the adBNST (light gray area) are shown unilaterally. ac: anterior commissure, ad: anterodorsal BNST, al: anterolateral BNST, av: anterior-ventral BNST, f: fornix, ic: internal capsule, ju: juxtacapsular BNST, LSv: lateral septum ventral division, lv: lateral ventricle, ov: oval nucleus of the BNST.

We have recently demonstrated that reduced activation of the CRF-R within the medial-posterior BNST (mpBNST) is necessary for the optimal expression of maternal behavior postpartum (Klampfl et al., 2014). Thus, in the present study we focused on the role of the CRF system in the anterior-dorsal BNST (adBNST) containing the oval, anterodorsal, anterolateral and juxtacapsular nuclei (Fig. 1) (Dong et al., 2001b) in regulating maternal and anxiety-related behavior in lactating rats. We acutely manipulated the CRF-R1 and -R2 in the adBNST with receptor-selective agonists and antagonists and assessed the effects on maternal care, maternal motivation, maternal aggression, and anxiety-related behavior during early lactation. Furthermore, we investigated the impact of acute intra-adBNST CRF-R manipulation on HPA axis activity under both basal and stressful conditions to determine whether the physiological changes observed might account for the changes in maternal behavior. Finally, we compared mRNA expression of the *Crf* gene and its receptors in virgin and lactating rats in the adBNST and additionally the anterior-ventral BNST (avBNST; Fig. 1), which appears to be also involved in the regulation of maternal behavior (Smith et al., 2012).

## 2 Materials & methods

### 2.1 Animals

Virgin female Sprague-Dawley rats (220–250 g; Charles River Laboratories, Sulzfeld, Germany) were kept under standard laboratory conditions (change of bedding once per week, RT 22 ± 2 °C, 55% relative humidity, 12:12 h light/dark cycle, lights on at 0600 h) with access to water and standard rat chow ad libitum. Females were mated with experienced stud males, and housed in groups of 3–4 until pregnancy day 18. For experiment 1, females underwent surgery on pregnancy day 18 and were single-housed thereafter to guarantee recovery and undisturbed delivery, and for experiment 2, 3 and 4, females were single-housed from pregnancy day 18 to guarantee undisturbed delivery as described recently (Bayerl et al., 2014, Klampfl et al., 2013). On the day of birth, litters were culled to eight pups of mixed sexes. For experiment 4, virgin females and lactating rats were treated identically, i.e., virgins were single-housed 7 days prior to brain collection, consistent with the single-housing period of the lactating rats. During the single-housing period (except the day before and the day of delivery), all rats were handled twice a day to reduce non-specific stress responses during the experiments.

For the maternal defense test, naïve virgin female Wistar rats (200–220 g, Charles River Laboratories) selected at random stages across the estrus cycle were used as intruders. Intruder rats were housed in a separate room to avoid olfactory recognition (Bosch, 2013).

All experiments were approved by the Committee on Animal Health and Care of the local government and conformed to international guidelines on the ethical use of animals. Efforts were made to minimize the number of rats used and their suffering.

### 2.2 Behavioral tests

All tests were analyzed online (maternal care, pup retrieval test (PRT), elevated plus-maze (EPM)) or from video recordings (maternal defense test; http://www.jwatcher.ucla.edu) by an experienced observer blind to the treatments.

#### 2.2.1 Maternal care

Maternal care was monitored according to an established protocol (Klampfl et al., 2014). Briefly, maternal care was observed before and after treatment, i.e., either substance infusion alone termed ‘non-stress condition’ or substance infusion followed by the maternal defense test, which is a psycho-social stressor (Neumann et al., 2001), termed ‘stress condition’ (Bayerl et al., 2014, Klampfl et al., 2014, Klampfl et al., 2013). The main parameter for the quality of maternal care was the occurrence of arched back nursing (ABN) (Bosch, 2011). Other behavioral parameters scored were hovering over the pups and blanket nursing posture, which together with ABN were counted as total nursing (quantity of maternal care). Pup retrieval/mouthing and licking/grooming were assessed as ‘other maternal behaviors’. Additionally, non-maternal (‘off-nest’) behaviors were scored, i.e., locomotion, self-grooming, and sleeping/resting. Data is shown in 30 min blocks before and after treatment infusion with a maximal count of 15 observations per block.

#### 2.2.2 Maternal motivation

The dams’ maternal motivation was tested in the PRT (Klampfl et al., 2014). Briefly, after 60 min of pup separation, the dam was placed in a plastic box (54 cm × 34 cm × 31 cm) and the number of retrieved pups within the 15-min testing period was counted.

#### 2.2.3 Maternal aggression

To assess maternal aggression, the maternal defense test was performed (Klampfl et al., 2014, Neumann et al., 2001). Briefly, the lactating dams (residents) were confronted with an unknown virgin female (intruder) in the dams’ home cage in the presence of their litter for 10 min. The following behavioral parameters were scored: total number of attacks, latency to first attack, lateral threat, keep down, and offensive upright as well as non-aggressive behaviors (for detailed description see (Bosch, 2013)).

#### 2.2.4 Anxiety-related behavior

Anxiety-related behavior was tested on the EPM (Klampfl et al., 2014, Pellow et al., 1985). Briefly, the dams were placed in the neutral zone of the maze and scored for 5 min. The percentage of time spent on the open arms versus all arms and of open arm entries versus all entries were taken as indicator of anxiety-related behavior. The number of closed arm entries was used to measure locomotion.

### 2.3 Experimental design

Schematic overviews of the timelines used for experiments 1–4 are shown in Fig. S1.

#### 2.3.1 Experiment 1: behavioral consequences of intra-adBNST manipulation of CRF-R1 or -R2 on maternal behavior

Experiment 1 was conducted according to an established protocol (Klampfl et al., 2014). Briefly, on PD 18, females were implanted bilaterally with 23 G guide cannula targeting the adBNST (−0.2 mm caudal, 3.0 mm lateral, 4.9 mm ventral to bregma (Paxinos and Watson, 1998); angle of 12.5°) under inhalation anesthesia (Isoflurane; Baxter Germany GmbH, Unterschleißheim, Germany) and semi-sterile conditions (Bosch et al., 2010). Substances were infused using a 27 G infusion cannula. Lactating rats received either (i) VEH (0.5 μl of sterile Ringer’s solution +4% DMSO; pH 7.4; B. Braun Melsungen, Melsungen, Germany), (ii) CRF-R1 agonist, human/rat CRF (1 μg/0.5 μl; Tocris Bioscience, Ellisville, Missouri, USA), (iii) CRF-R1 specific antagonist, CP-154,526 (12 μg/0.5 μl; Tocris Bioscience), (iv) CRF-R2 specific agonist, hUcn 3 (stresscopin; 3 μg/0.5 μl; Phoenix Pharmaceuticals, Karlsruhe, Germany), or (v) CRF-R2 specific antagonist (astressin-2B; 4 μg/0.5 μl; Sigma–Aldrich, Steinheim, Germany). Stresscopin has previously been shown to be highly selective for CRF-R2, and is without effect on CRF-R1 expressing cells (Hsu and Hsueh, 2001). Doses and the lag time between the infusion and behavioral testing were chosen based on previous studies (D’Anna and Gammie, 2009, D’Anna et al., 2005, Gammie et al., 2004, Klampfl et al., 2014, Sahuque et al., 2006). On each test day, the lactating dams received a single acute bilateral infusion either 10 min (VEH, CRF-R1 agonist, CRF-R1 antagonist, CRF-R2 antagonist) or 25 min (CRF-R2 agonist) prior to the tests. Each animal received the same treatment on every testing day as assigned on LD 1. Importantly, the repeated infusions of the CRF-R1 or -R2 (ant-) agonists separated by 48 h intervals are not expected to result in receptor (de)sensitization (Hauger et al., 2009, Klampfl et al., 2014, Spiess et al., 1998).

Maternal care was observed under non-stress conditions (LD 1) and stress conditions (LD 7) in the home cage. Under non-stress conditions, dams were observed from 0800 h to 0900 h, received an infusion and were observed immediately afterwards for 120 min. Additionally, dams were observed again 5 h after infusion, from 1400 h to 1500 h, to assess potential long-lasting effects of drug treatment. Under stress conditions, dams were observed from 0800 h to 0900 h, moved to the test room, and infused at 1000 h. Dams were tested in the maternal defense test, and immediately afterwards moved back to the observation room, where maternal care was observed for another 60 min in order to assess the effects of the preceding stressor on maternal care. Additionally, maternal motivation (LD 3), anxiety-related behavior (LD 5), and maternal aggression (LD 7) were tested as described above. All tests were performed between 0800 h and 1500 h.

#### 2.3.2 Experiment 2: effect of intra-adBNST manipulation of CRF-R1 or -R2 on basal and stress-induced HPA axis activity

On LD 1, separate groups of rats were bilaterally implanted with a local guide cannula targeting the adBNST as described in Section 2.3.1. In order to determine HPA axis activity following intra-adBNST CRF-R manipulations, dams were also fitted with a jugular vein catheter for repeated blood sampling in conscious, freely moving rats, as previously described (Bosch et al., 2007).

On LD 6 at 0800 h, the catheters were connected to a sampling syringe filled with heparinized saline (0.6%; Ratiopharm, Ulm, Germany). After 90 min of habituation, blood samples (0.25 ml) were collected into EDTA-coated tubes and stored on ice. The first two samples were withdrawn 30 min apart under basal conditions. Immediately afterwards, the dams were infused bilaterally into the adBNST with VEH, CRF-R1 agonist, CRF-R2 agonist, CRF-R1 antagonist, or CRF-R2 antagonist (drug details and doses are given in Section 2.3.1). Further blood samples were collected 10 and 30 min after the treatment to assess the effect of specific CRF-R activation/inhibition on HPA axis activity. Thirty minutes after the drug infusion, dams were exposed to the maternal defense test for 10 min (see Section 2.2.3). Additional blood samples were collected 5, 15, and 60 min after termination of the stressor exposure. All blood samples were immediately replaced with 0.9% sterile saline. Samples were centrifuged for 15 min at 4 °C (5000 rpm) and stored at −20 °C until further processing.

Plasma ACTH (sensitivity: 0.22 pg/ml; intra-assay and inter-assay coefficients of variation were ≤7.1%) and CORT (sensitivity: <0.56 ng/ml; intra-assay and inter-assay coefficients of variation ≤6.35%) concentrations were measured using commercially available ELISA kits (IBL International GmbH, Hamburg, Germany).

#### 2.3.3 Experiment 3: behavioral consequences of HPA axis activation on maternal care

In order to investigate a potential influence of HPA axis activation on maternal care, a separate cohort of lactating rats was fitted with a jugular vein catheter for i.v. injection on LD 1 (for details see Section 2.3.2).

On LD 6, the catheters were connected at 0800 h and the dams were left undisturbed for 120 min. Between 0900 h and 1000 h, the occurrence of maternal care was observed under basal conditions as described in Section 2.2.1. At 1000 h, dams were acutely infused i.v. via the catheter with either 100 μl 0.9% sterile saline (VEH) or 100 μl ACTH_(1–39)_ (mouse, rat; 0.1 μg/ml; Bachem, Bubendorf, Switzerland) and observed for another 120 min to assess any potential influences of ACTH infusion (and hence acutely elevated CORT levels) on maternal care.

In order to verify a physiological CORT response following acute ACTH infusion, the catheters of the same rat cohort were connected on LD 7 at 0800 h and blood samples were withdrawn as described in Section 2.3.2. The first two samples were withdrawn 30 min apart under basal conditions. Immediately afterwards, the dams were infused i.v. via the catheter according to LD 6. In addition, a third naïve group was subjected to a 10-min maternal defense test in order to compare the endogenous HPA axis response (Neumann et al., 2001) to the ACTH-induced stress response. Further blood samples were collected 5, 15 and 60 min after injection/stressor exposure. All blood samples were treated and analyzed as described in Section 2.3.2. The dose of ACTH was based on a preliminary study (Perani et al., 2015).

#### 2.3.4 Experiment 4: expression of *Crf* and *Crfr* mRNA in the anterior BNST of virgin and lactating rats

In order to compare the effect of reproductive status on *Crf*, *Crfr1* and *Crfr2* mRNA expression in the adBNST, a separate cohort of naïve, untreated virgin and lactating rats was killed under basal conditions in the morning of LD 4 or equivalent in virgin rats, i.e., after 7 days of single-housing. The brains were rapidly removed, flash frozen on dry ice, and stored at −20 °C until further processing (see Section 2.4).

### 2.4 *In situ* hybridization for *Crf* and *Crfr* mRNA expression

Frozen brains were sectioned at 16 μm using a cryostat (Model CM3050S Leica Microsystems GmbH, Nussloch, Germany), mounted on polysine slides, and stored at −20 °C until further processing.

*Crf* mRNA *in situ* hybridization was conducted following an established protocol using a highly-specific 48-base, 3′-end ^35^S-labeled oligonucleotide probe (De Vries et al., 1994, Wang et al., 1994). *Crfr1* and *Crfr2* mRNA *in situ* hybridization was performed using an established protocol with previously described cRNA probes for *Crfr1* and *Crfr2* (Brunton et al., 2011, Brunton et al., 2009). Autoradiograms of the adBNST and avBNST (Bregma +0.36 mm to −0.4 mm (Paxinos and Watson, 1998)) were analyzed with Image J (V 1.46, NIH image software) as described previously (Brunton et al., 2011, Klampfl et al., 2013). Measurements were made bilaterally over six sections per rat. Brain sections hybridized with ^35^S-UTP-labeled cRNA sense probes (negative controls) showed no signal above background.

### 2.5 Histology

To verify the correct placements of local cannula within the adBNST, rats were decapitated and brains were infused with 0.5 μl of ink (Pelikan Ink 4001, Hanover, Germany; diluted 1:20 in Ringer’s solution), removed, flash frozen, cut in 40 μm coronal sections, and slide mounted. Ink diffusion was only detected within the adBNST as assessed according to our previous study (Klampfl et al., 2014). Additionally, slides were stained via quick Nissl staining to locate the tip of the infusion cannula (Fig. 1, black dots). Only rats with correctly located cannula and properly restricted ink diffusion were included in the statistical analysis.

### 2.6 Statistical analysis

Behavioral and physiological data were analyzed using either one-way analysis of variance (ANOVA; factor: treatment) or two-way RM ANOVA (factors: time × treatment) followed by Fisher’s LSD post hoc test for parametric data, and Kruskal–Wallis *H* test for non-parametric data. *In situ* hybridization data was analyzed using independent *t*-tests. While typically tests were performed over all groups, only comparisons to VEH-treated rats or to basal values are shown. For all tests, the software package SPSS 19.0 (IBM, Armonk, New York, USA) was used. Data are presented as means ± SEM and significance was accepted at *p* ≤ 0.05.

## 3 Results

### 3.1 Experiment 1: consequences of intra-adBNST manipulation of CRF-R1 or -R2 on maternal behavior and anxiety

#### 3.1.1 Maternal care under non-stress conditions

##### 3.1.1.1 ABN

**Fig. 2:**
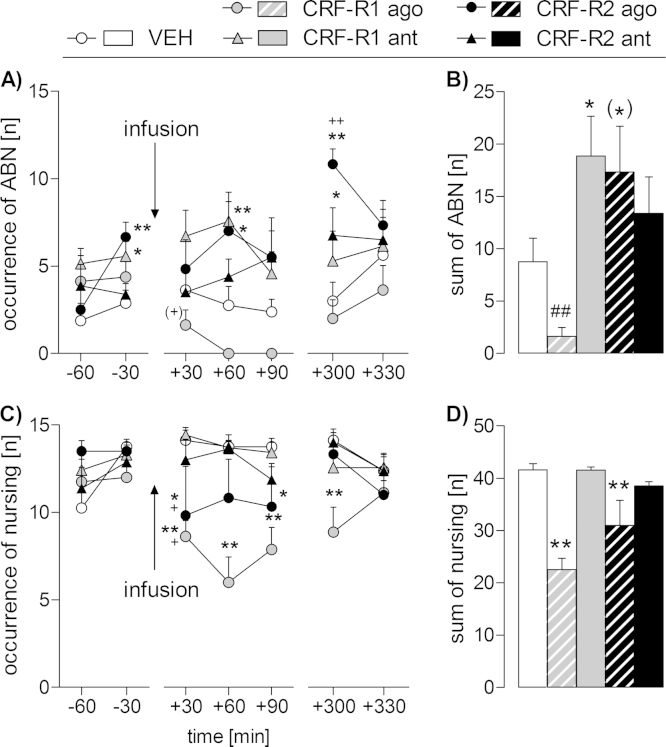
Effect of intra-adBNST CRF-R1 or -R2 specific agonist (ago) or antagonist (ant) treatment on maternal care of lactating dams under non-stress conditions on LD 1. Arched back nursing (ABN; A, B) and total nursing (C, D) were scored for 60 min before, for 90 min after infusion and for additional 60 min following a 5 h delay. The data collected during the 90 min period after the drug infusion are summed up and shown on the right side (B, D). Dams received an acute bilateral infusion of either (i) vehicle (VEH), (ii) a CRF-R1 ago (CRF), (iii) a CRF-R1 ant (CP-154,526), (iv) a CRF-R2 ago (stresscopin), or (v) a CRF-R2 ant (astressin-2B) into the adBNST. Data is presented as group means + SEM. *n* = 6–8 rats per group. **p ≤ 0.01, *p ≤ 0.05 versus VEH-treated group; ++*p* ≤ 0.01, +*p* ≤ 0.05, (+)*p* = 0.07 versus *t*-30 min time-point of the same group (time course: two-way RM ANOVA; sum: one-way ANOVA), ##*p* ≤ 0.01 versus VEH (independent *t*-test).

Differences in ABN were found depending on time (two-way RM ANOVA; F_(6,192)_ = 3.14, *p *< 0.01; Fig. 2A) and treatment (F_(4,32)_ = 6.62, *p *< 0.01), with a significant interaction between these factors (F_(24,192)_ = 1.89, *p *= 0.01). Before the infusion, dams treated with the CRF-R1 antagonist (*p *= 0.03) or CRF-R2 agonist (*p *< 0.01) displayed more ABN at *t*-30 min compared with the VEH group. Immediately after the CRF-R1 agonist infusion, ABN tended to be reduced compared with the *t*-30 min time-point (*p *= 0.07). Nevertheless, at *t *+ 60 and *t *+ 90 min ABN was essentially abolished in the CRF-R1 agonist-treated rats. Furthermore, ABN was significantly greater at *t* + 60 min in the CRF-R1 antagonist (*p *< 0.01) and CRF-R2 agonist (*p *= 0.02) groups compared with the VEH-treated group. In the afternoon, both the CRF-R2 agonist (*p *< 0.01) and CRF-R2 antagonist (*p *= 0.04) groups exhibited significantly greater ABN compared with the VEH-treated group.

When summing up the occurrence of ABN after the drug infusion (i.e., from *t *+ 30 min until *t *+ 90 min), significant differences were found between the groups (one-way ANOVA, F_(4,32)_ = 5.31, *p *= 0.02; Fig. 2B). The CRF-R1 antagonist significantly increased ABN (*p *= 0.02), and there was a trend toward an increase in ABN in the CRF-R2 agonist-treated group (*p *= 0.06). Additionally, the CRF-R1 agonist markedly decreased ABN following the infusion compared with the VEH-treated rats (independent *t*-test; *t*_(14)_ = 2.95, *p *= 0.01).

##### 3.1.1.2 Total nursing

Differences in nursing were found dependent on treatment (two-way RM ANOVA; F_(4,32)_ = 12.18, *p* < 0.01; Fig. 2C) and a significant interaction was detected between treatment and time (F_(24,192)_ = 2.20, *p* < 0.01). Before the drug infusion, no differences were detected in total nursing. After the infusion, dams treated with the CRF-R1 agonist showed significantly less nursing at *t* + 30 min compared with the previous time-point (*p *= 0.02), as well as at *t* + 30 min, *t* + 60 min, *t* + 90 min and *t* + 300 min (*p* < 0.01 in each case) compared with the VEH-treated rats. Dams treated with the CRF-R2 agonist showed reduced nursing at *t* + 30 min compared with the previous time-point and with the VEH-treated dams (*p *= 0.03 in each case) as well as at *t* + 90 min compared with VEH-treated dams (*p *= 0.05). When summing up the occurrence of total nursing after the infusion, significant differences were found between the groups (one-way ANOVA; F_(4,32)_ = 15.83, *p* < 0.01; Fig. 2D). Dams treated with the CRF-R1 agonist (*p* < 0.01) or CRF-R2 agonist (*p* < 0.01) showed a significant reduction in nursing compared with those treated with VEH.

There was no significant difference in any other maternal behaviors measured (i.e., home cage pup retrieval/mouthing, licking/grooming) on LD1 (data not shown); however, differences in off-nest behavior were detected (Supplementary data and Table S1).

#### 3.1.2 Maternal care under stress conditions

##### 3.1.2.1 ABN

**Fig. 3:**
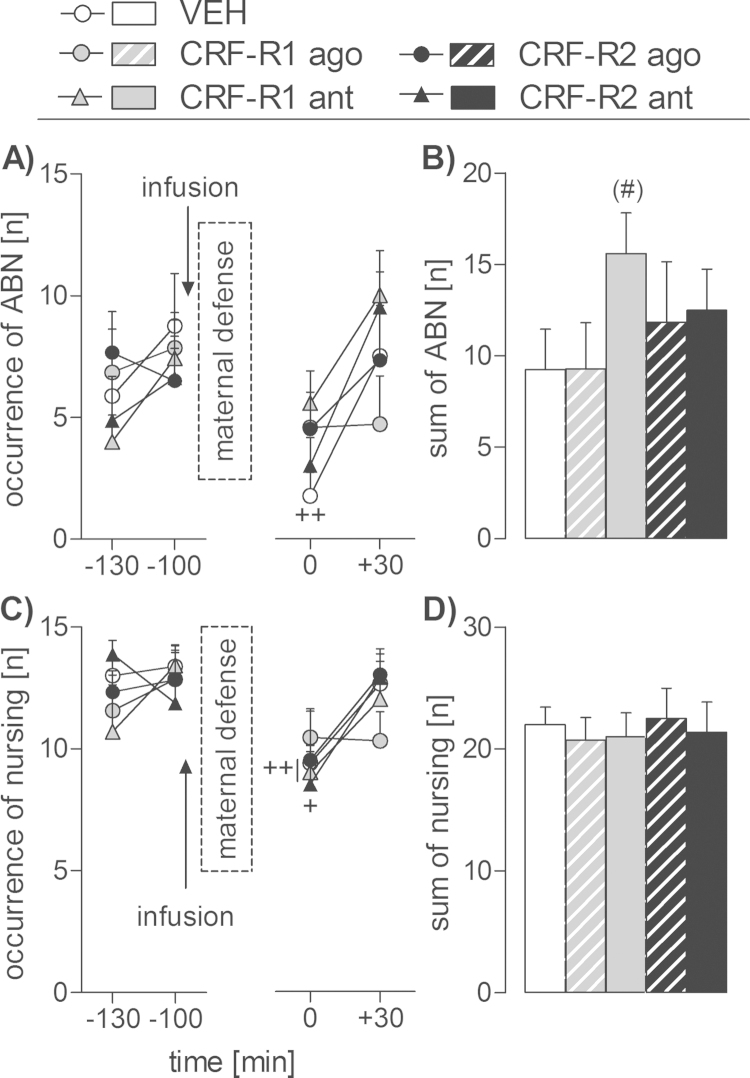
Effect of intra-adBNST CRF-R1 or -R2 specific agonist (ago) or antagonist (ant) treatment on maternal care of lactating dams under stress conditions on LD 7. Arched back nursing (ABN; A, B) and total nursing (C, D) were scored for 60 min before and after infusion combined with the maternal defense test. The 60 min afterwards are summed up and shown on the right side (B, D). Dams received an acute bilateral infusion of either (i) vehicle (VEH), (ii) a CRF-R1 ago (CRF), (iii) a CRF-R1 ant (CP-154,526), (iv) a CRF-R2 ago (stresscopin), or (v) a CRF-R2 ant (astressin-2B) into the adBNST. Data presented are group means + SEM. *n* = 6–8 rats per group. ++*p* ≤ 0.01, +*p* ≤ 0.05 versus *t*-30 min time-point of the same group (two-way RM ANOVA); (#)*p* ≤ 0.06 versus VEH (independent *t*-test).

Time-dependent differences in ABN were found under stress conditions (two-way RM ANOVA; F_(3,93)_ = 6.56, *p* < 0.01; Fig. 3A). ABN was significantly reduced in the VEH group immediately after termination of the stressor exposure (*t* 0 min compared to *t*-100 min; *p* < 0.01). When summing up ABN after the drug infusion and the maternal defense test, no significant differences were detected. However, an independent *t*-test revealed a strong trend toward increased ABN in the CRF-R1 antagonist-treated dams compared with the VEH-treated dams (*t*_(13)_ = −2.00, *p *= 0.06; Fig. 3B).

##### 3.1.2.2 Total nursing

Differences in total nursing were found depending on time (two-way RM ANOVA; F_(3,93)_ = 10.05, *p* < 0.01; Fig. 3C). Total nursing was reduced in the VEH- (*p *= 0.01), CRF-R1 antagonist- (*p* < 0.01) and CRF-R2 antagonist-treated groups (*p *= 0.03) at *t* 0 min compared to *t*-100 min. When summing up nursing after the infusion and the maternal defense test, no significant differences were found (Fig. 3D). Furthermore, no significant differences in home cage pup retrieval/mouthing and licking/grooming were found (data not shown); however, differences were detected in non-maternal behaviors (Supplementary data and Table S2).

#### 3.1.3 Maternal motivation

None of the treatments affected pup retrieval behavior (data not shown).

#### 3.1.4 Maternal aggression

None of the treatments affected aggressive or non-aggressive behaviors in the maternal defense test (data not shown).

#### 3.1.5 Anxiety-related behavior

None of the treatments had any significant effect on the percentage of time spent on the open arms or on the number of entries into the open or closed arms of the EPM (data not shown).

### 3.2 Experiment 2: effect of intra-adBNST manipulation of CRF-R1 or -R2 on HPA axis activity under basal and stressful conditions

**Fig. 4:**
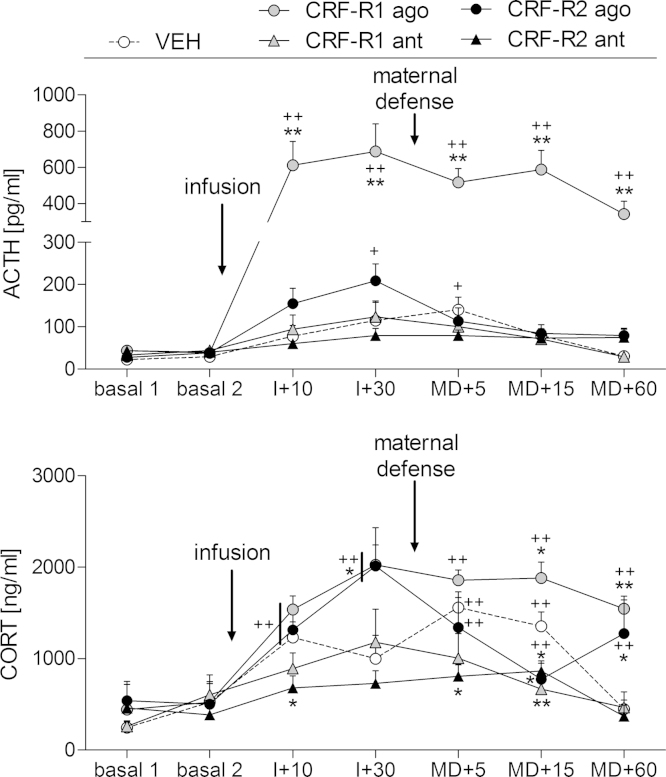
Effect of intra-adBNST CRF-R1 or -R2 specific agonist (ago) or antagonist (ant) treatment on plasma ACTH and corticosterone (CORT) concentrations. ACTH and CORT were measured under basal conditions (basal 1, basal 2), 10 (I + 10) and 30 min (I + 30) after infusion, and 5 (MD + 5), 15 (MD + 15), and 60 min (MD + 60) after the maternal defense test. Dams received an acute bilateral infusion of either (i) vehicle (VEH), (ii) a CRF-R1 ago (CRF), (iii) a CRF-R1 ant (CP-154,526), (iv) a CRF-R2 ago (stresscopin), or (v) a CRF-R2 ant (astressin-2B) into the adBNST. Data presented are group means + SEM. *n* = 5–8 rats per group. ++*p* ≤ 0.01, +*p* ≤ 0.05 versus basal 2 of the same group; **p ≤ 0.01, *p ≤ 0.05 versus VEH (two-way RM ANOVA).

There was a significant effect of time (two-way RM ANOVA; F_(1.94,156)_ = 24.04, *p* < 0.01; Fig. 4 top) and treatment (F_(4,26)_ = 16.11, *p* < 0.01) and a significant interaction between time and treatment (F_(7.8,156)_ = 9.26, *p* < 0.01; corrected after Greenhouse-Geisser) on plasma ACTH concentrations. Dams treated with either VEH, the CRF-R1 or the CRF-R2 agonist showed significant changes in ACTH levels over time (Fig. 4, top). In the VEH-treated dams, no changes in basal plasma ACTH were found following the infusion. However, after exposure to the maternal defense test, plasma ACTH levels increased transiently at MD + 5 compared with basal 2 (*p *= 0.02). In the CRF-R1 agonist-treated dams, plasma ACTH was significantly increased within 10 min of the infusion and remained elevated from basal levels at I + 30 (*p* < 0.01 in each case). Maternal defense had no further effect on ACTH secretion in the CRF-R1 agonist treated dams, with levels remaining elevated across the blood sampling period (Fig. 4, top). In the CRF-R2 agonist-treated dams, ACTH secretion was increased at I + 30 compared with basal 2 levels (*p *= 0.05), however, there was no further effect of stress; on the contrary, plasma ACTH concentrations declined to levels not different from basal levels after the maternal defense test (Fig. 4, top). No significant changes in ACTH secretion were detected in either the CRF-R1 or CRF-R2 antagonist-treated dams.

When comparing all the different treatment groups, no differences were found before the infusion, whereas the CRF-R1 agonist infusion resulted in significantly greater plasma ACTH concentrations at all of the time-points after the infusion compared with the VEH-treated group (*p* < 0.01 in each case).

There was a significant effect of time (two-way RM ANOVA; F_(3.8,186)_ = 21.83, *p* < 0.01; Fig. 4 bottom) and treatment (F_(4,31)_ = 8.46, *p* < 0.01) and a significant interaction between the factors (F_(15.3,186)_ = 2.71, *p* < 0.01; corrected after Greenhouse-Geisser) on plasma CORT concentrations. All mothers, except the CRF-R1 antagonist-treated dams, showed significant changes in CORT secretion over time. In the VEH-treated dams, plasma CORT concentrations increased from basal levels after the infusion at I + 10 (*p* < 0.01), plateaued and were further increased after the maternal defense test at MD + 5 and MD + 15 (*p* < 0.01 versus basal 2 in each case), before returning to basal levels at MD + 60. Infusion of the CRF-R1 agonist significantly increased plasma CORT concentrations compared with basal levels (*p* < 0.01). There was no further increase in CORT secretion after maternal defense in the CRF-R1 agonist-treated dams; however, plasma CORT remained significantly elevated above basal levels 60 min after the stressor exposure. In the CRF-R2 agonist-treated dams, CORT secretion was significantly increased after the infusion at I + 10 and I + 30 (*p* < 0.01 in each case) compared with basal 2 levels. However, following stressor exposure, plasma CORT concentrations were decreased at MD + 5 in the CRF-R2 agonist-treated dams but were still significantly higher than basal levels (*p* < 0.01). No differences were detected at MD + 15 compared with basal levels in the CRF-R2 agonist-treated dams, however, CORT secretion increased again at MD + 60 (*p* < 0.01 versus basal 2). In the CRF-R1 antagonist-treated dams, CORT concentrations did not differ from basal at any time-point. The CRF-R2 antagonist-treated dams showed increased plasma CORT concentrations compared with basal levels only at MD + 15 (*p* < 0.01 versus basal 2).

Comparison of the different treatment groups at the various time-points demonstrated that all dams showed differences in plasma CORT concentrations after drug infusion compared with VEH-treated rats. Infusion of the CRF-R1 agonist resulted in a significantly greater increase in CORT secretion at I + 30 (*p* < 0.01), with plasma CORT concentrations remaining elevated at MD + 15 (*p *= 0.02) and MD + 60 (*p* < 0.01). The CRF-R1 antagonist prevented the stress-induced increase in CORT secretion at MD + 15 observed in the VEH-treated dams (*p* < 0.01 versus VEH). Infusion of the CRF-R2 agonist significantly increased circulating CORT concentrations at I + 30 (*p *= 0.02); however, after exposure to the maternal defense test, CORT secretion decreased at MD + 15 (*p *= 0.01), before rising again at MD + 60 (*p *= 0.01). Infusion of the CRF-R2 antagonist had no significant effect on basal CORT secretion; however, it did prevent the stress-induced increase in CORT secretion observed in the VEH-treated dams (I + 10: *p* = 0.02; MD + 5: *p* = 0.02; MD + 15: *p* = 0.03 versus VEH).

### 3.3 Experiment 3: behavioral consequences of HPA axis activation on maternal care

#### 3.3.1 Maternal care after i.v. VEH or ACTH infusion

On LD 6, infusion of VEH or ACTH did not alter any parameter of maternal care (Supplementary data and Table S3).

#### 3.3.2 Plasma ACTH and CORT concentrations following i.v. ACTH infusion or stressor exposure

**Fig. 5:**
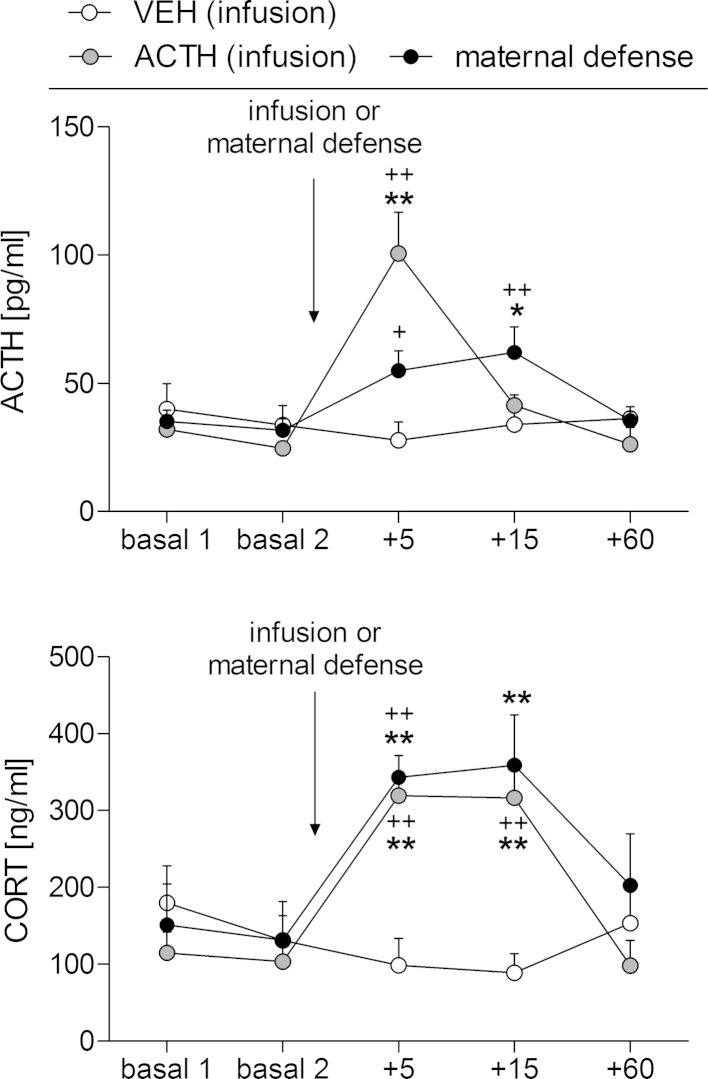
Effect of i.v. ACTH infusion or exposure to the maternal defense (MD) test on plasma ACTH and corticosterone (CORT) concentrations. ACTH and CORT were measured under basal conditions (basal 1, basal 2) and 5, 15, and 60 min after infusion or exposure to the MD test. Dams received an acute i.v. infusion of vehicle (VEH) or ACTH_(1–39)_. Data presented are group means  +  SEM. *n* = 6–9 rats per group. **p ≤ 0.01, *p ≤ 0.05 versus VEH; ++*p* ≤ 0.01, +*p* ≤ 0.05 versus basal 2 of the same group (two-way RM ANOVA).

There was a significant effect of time (two-way RM ANOVA; F_(4,64)_ = 14.49, *p* < 0.01) but not of treatment, and a significant interaction between time and treatment on plasma ACTH concentrations (F_(8,64)_ = 10.54, *p* < 0.01; Fig. 5 top). As expected, the ACTH infusion significantly increased plasma ACTH concentrations at *t* + 5 min compared with basal levels and with the VEH-treated group (*p* < 0.01 in each case); with levels returning to basal by *t* + 15 min. The maternal defense test significantly increased ACTH secretion at *t* + 5 min (*p *= 0.04 versus basal 2) and *t* + 15 min (*p* < 0.01 versus basal 2, *p *= 0.01 versus VEH).

There was a significant effect of time (two-way RM ANOVA; F_(4,80)_ = 10.44, *p* < 0.01) but not of treatment, and a significant interaction between time and treatment on plasma CORT concentrations (F_(8,80)_ = 6.15, *p* < 0.01; Fig. 5 bottom). Plasma CORT concentrations in both the ACTH-treated dams and the dams exposed to the maternal defense test were significantly increased at *t* + 5 min and *t* + 15 min compared with basal concentrations (*p* < 0.01 in each case), before declining to basal levels at *t* + 60 min. Moreover, CORT secretion was significantly greater in the ACTH-treated and maternal defense-exposed dams at *t* + 15 min compared with the VEH-treated rats (*p* < 0.01 in each case).

### 3.4 Experiment 4: *Crf* and *Crfr* mRNA expression in the anterior BNST of virgin and lactating rats

**Fig. 6:**
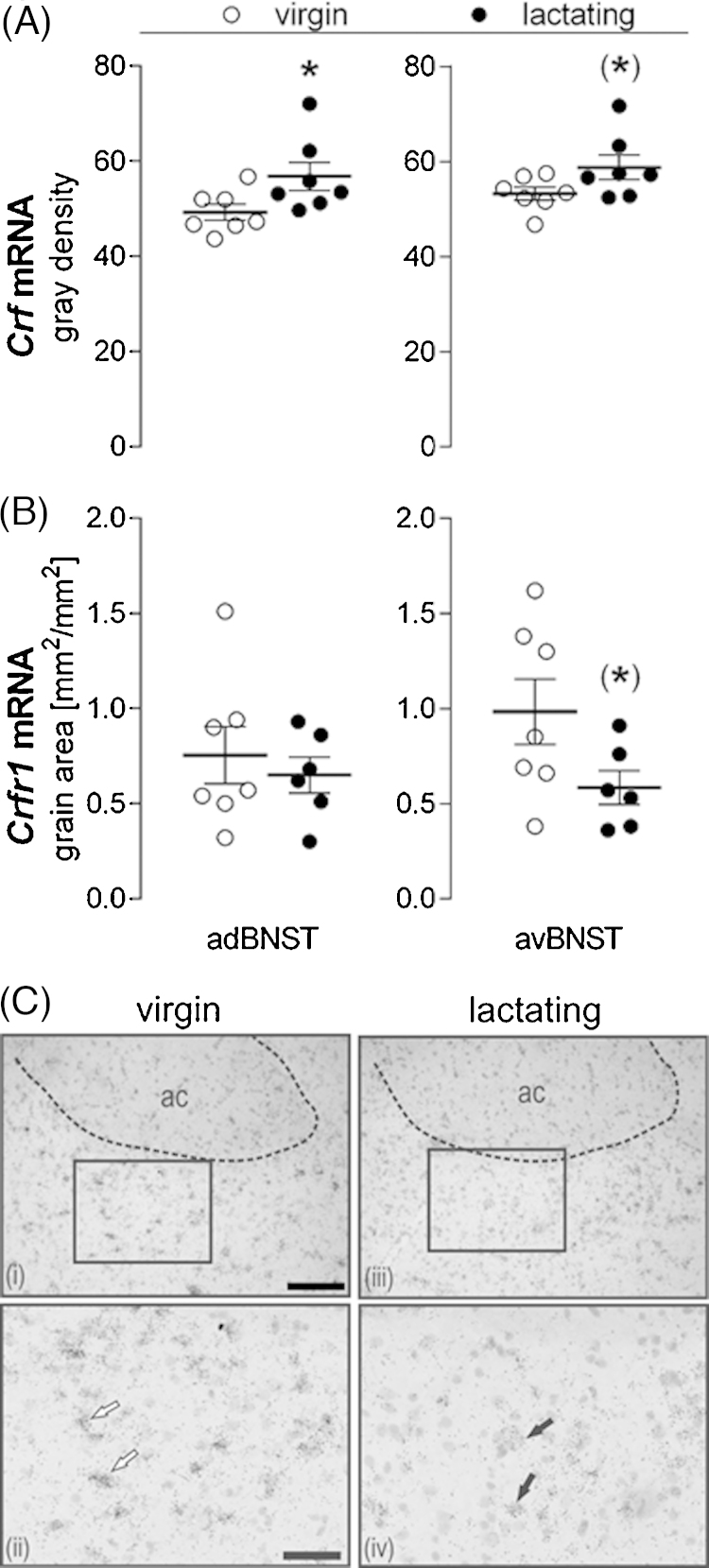
*Crf* and *Crfr1* mRNA expression in the adBNST and avBNST of virgin and lactating rats under non-stress conditions. Quantification of (A) *Crf* mRNA and (B) *Crfr1* mRNA presented as gray density (A) or grain area (mm^2^/mm^2^; B). (C) Representative brightfield photomicrographs of *Crfr1* mRNA expression in the avBNST from (i) a virgin and (iii) a lactating rat, (ii) and (iv) are high power images of the area delineated by the box in (i) and (iii), respectively. Arrows indicate examples of positively hybridized cells from a virgin (open arrows) and a lactating rat (filled arrows). ac, anterior commissure; scale bars: 200 μm, upper panels; 100 μm, lower panels. Data presented are group means ± SEM. *n* = 6–7 rats per group. *p < 0.05, (*)*p* = 0.07 (independent *t*-test).

*Crf* mRNA expression was significantly greater in the adBNST (independent *t*-test; *t*_(12)_ = −2.20, *p *= 0.04) and tended to also be greater in the avBNST (*t*_(12)_ = −1.92, *p *= 0.07) of lactating rats compared with virgin rats (Fig. 6A).

*Crfr1* mRNA expression was similar in the adBNST, though there was a tendency for reduced expression in the avBNST (independent *t*-test; *t*_(11)_ = 1.96, *p *= 0.07) of lactating rats compared with virgin rats (Fig. 6B, c). *Crfr2* mRNA expression was not detected in the adBNST or the avBNST of virgin or lactating rats, consistent with previous reports in males and virgin females (Van Pett et al., 2000).

## 4 Discussion

The activity of the maternal brain’s CRF system is attenuated to ensure appropriate maternal behavior (Gammie et al., 2004, Klampfl et al., 2013, Pedersen et al., 1991). The BNST represents a major regulatory site, as activation of predominantly CRF-R2 in the mpBNST results in maternal neglect (Klampfl et al., 2014). Here, we provide evidence that the CRF system in the adBNST exerts distinct and more selective roles in modulating maternal behavior. Activation of CRF-R1 in the adBNST reduced maternal care but not maternal motivation, aggression or anxiety in lactating rats. Importantly, these effects appear to be mediated independent of simultaneous HPA axis activation.

Under basal conditions, CRF-R1 activation in the adBNST impaired nursing, suggesting CRF-R1 stimulation during lactation leads to neglect of the offspring. CRF-R2 activation in the adBNST resulted in increased ABN after a 5 h delay, which probably compensates for the initial decline in nursing. In contrast to the current study, CRF-R2 activation in the mpBNST is detrimental to ABN (Klampfl et al., 2014), indicating that potential diffusion from the adBNST to the mpBNST is unlikely. Moreover, we found that CRF-R2 activation decreased the occurrence of total nursing implying that the CRF-R2 agonist-treated dams nurse less promptly after the infusion, which was later reversed. In accordance, CRF-R2 are generally considered to aid stress recovery upon activation (Bale et al., 2002, Bale and Vale, 2004, Reul and Holsboer, 2002) and genetic deletion of CRF-R2 impairs maternal aggression in lactating mice (D’Anna et al., 2008). In contrast, CRF-R2 activation in the mpBNST of lactating rats is detrimental to maternal behavior (Klampfl et al., 2014).

Following stressor exposure, CRF-R1 inhibition tended to increase ABN, indicating that hyper-activation of adBNST CRF-R1 might be detrimental to maternal care under stressful conditions. Interestingly, this is in contrast to the mpBNST (Klampfl et al., 2014) where inhibition of CRF-R2 has similar behavioral effects as inhibition of CRF-R1 in the adBNST suggesting that maternal care is differentially regulated by CRF-R in a site specific-manner: in the anterior BNST CRF-R1 seems to be the dominant receptor regulating maternal care, whereas in the posterior BNST CRF-R2 predominate in modulating maternal care and aggression as well as anxiety-related behavior (Klampfl et al., 2014).

Maternal motivation was unaffected by any treatment in the adBNST, similar to the mpBNST (Klampfl et al., 2014). This was anticipated as only the avBNST interconnects with the medial preoptic area, a main site regulating maternal motivation (Numan and Woodside, 2010). Hence, the ad/mpBNST CRF system is evidently not involved in maternal motivation in lactating rats.

Maternal aggression was not affected by any CRF-R manipulation in the adBNST, in contrast to the mpBNST where CRF-R2 activation abolishes attacking behavior and aggressive encounters (Klampfl et al., 2014). Unlike the adBNST, the mpBNST highly expresses *Crfr2* mRNA (Klampfl et al., 2014) and is implicated in defensive behaviors (Bosch, 2013, Dong and Swanson, 2004). Given the CRF-R2 subtype is involved in mediating maternal aggression (D’Anna and Gammie, 2009, Klampfl et al., 2014) and *Crfr2* mRNA was not detected in the adBNST, a lack of effect may underlie the behavioral differences resulting from CRF-R manipulation in the ad- and mpBNST.

Anxiety-related behavior was unaffected by CRF-R manipulation in the adBNST. This was unexpected since the BNST CRF system regulates anxiety (Klampfl et al., 2014, Sahuque et al., 2006, Walker et al., 2003) and CRF-R2 inhibition in the anterolateral BNST is anxiogenic in male Fisher-344 rats (Tran et al., 2014). This discrepancy may be a consequence of sex-specific anxiety regulation (Klampfl et al., 2014, Toufexis, 2007), abnormal HPA axis functioning and/or extremely low anxiety levels in male Fisher-344 rats (Tran et al., 2014). Furthermore, it is possible that any effect on anxiety-related behavior mediated via CRF-R1 or -R2 in the adBNST is masked due to the opposing influence of the oval nucleus and the anterodorsal nucleus (which were both targeted by the infusions) on anxiety-related behavior (Kim et al., 2013). Thus, given the lack of effect of CRF-R manipulation in the adBNST and a strong anxiolytic effect of CRF-R inhibition in the mpBNST of lactating rats (Klampfl et al., 2014), we propose that the anxiety-mediating effects of CRF might be restricted to the mpBNST during lactation.

The BNST is vital for HPA axis regulation (Crestani et al., 2013) as well as acutely (Pereira et al., 2015) and chronically (Brummelte and Galea, 2010a) elevated CORT levels are detrimental to maternal care. Here, acute activation of CRF-R1 increased plasma ACTH and CORT, while activation of CRF-R2 increased ACTH to a lesser extent than CRF-R1 activation and affected CORT in a stress-dependent context, indicating differential regulation of the HPA axis by the two receptor subtypes. Only a small proportion of neurons in the adBNST, i.e., those in the oval nucleus (Dong et al., 2001b), directly project to the PVN, however it is unclear if this pathway plays an important role in mediating the modulatory actions of CRF-R1/R2 activation in the adBNST on HPA axis activity. An alternative explanation is that the GABAergic projection from the adBNST to the avBNST plays a more important role. Given avBNST neurons send GABAergic projections to the PVN CRF neurons (Crestani et al., 2013, Turesson et al., 2013), activation of adBNST CRF-R1 might activate the HPA axis via disinhibition of the GABAergic input to the hypothalamus from the avBNST. As CRF-R1 activation stimulated ACTH secretion to a greater extent than CRF-R2 activation, and CORT secretion was similarly induced by activation of both receptors, a dissociation of the hypothalamus/pituitary and adrenal glands also seems feasible. Thus, following CRF-R2 activation, CORT secretion might be stimulated via activation of the sympathetic nervous system, rather than driven by the pituitary. This is supported by studies showing projections from the adBNST to autonomic brainstem nuclei (Dong et al., 2001b, Herman et al., 2003, Spencer et al., 2005). Interestingly, CRF-R2 agonist-induced CORT secretion was markedly reduced following exposure to stress, thus it appears that CRF-R2 may switch function depending on the presence or absence of stress. Under basal conditions, CRF-R2 seem to act in concert with CRF-R1, while under stress conditions, CRF-R2 assume their well-documented role in terminating the stress response (Bale et al., 2002, Bale and Vale, 2004). Our data clearly demonstrates that CRF-R manipulation in the adBNST strongly influences HPA axis activity, which could potentially affect maternal care via CORT secretion (Brummelte and Galea, 2010a, Pereira et al., 2015). Importantly, an acute rise in circulating ACTH and the concomitant increase in peripheral CORT did not impact maternal care, indicating that the behavioral changes observed following intra-adBNST CRF-R manipulation are likely centrally mediated, rather than a result of activation of the peripheral stress axis. It is unlikely that the dose of ACTH was too low to induce behavioral changes given the ACTH infusion and maternal defense, which typically reduces maternal care (Bayerl et al., 2014, Klampfl et al., 2014, Klampfl et al., 2013), increased CORT concentrations to similar levels.

With respect to peripartum adaptations, *Crf* mRNA expression in the adBNST was greater in lactating rats than in virgins, while *Crfr1* mRNA expression was not different. Interestingly, similar findings were reported on the mpBNST (Klampfl et al., 2014, Walker et al., 2001). Thus, it is likely that hypo-activation of CRF-R1 required for adequate maternal behavior is achieved by down-regulation of other CRF-family members. We did not detect *Crfr2* mRNA expression in the adBNST or avBNST, consistent with findings in male and virgin females (Van Pett et al., 2000). Thus, the behavioral and physiological changes observed after adBNST CRF-R2 manipulation may have been mediated via an adjacent brain region e.g., the lateral septum, which abundantly expresses *Crfr2* mRNA (Potter et al., 1994). In particular, the ventral septal region exhibits bidirectional connections with the PVN (Risold and Swanson, 1997) and might therefore account for the HPA axis activation following CRF-R2 manipulation. We can exclude diffusion to the mpBNST due to the different behavioral effects of CRF-R2 activation in the adBNST versus the mpBNST (Klampfl et al., 2014). Moreover it is unlikely that the CRF-R2 agonist is acting on CRF-R1, given it is highly selective for CRF-R2 (Hsu and Hsueh, 2001, Lim et al., 2005). Although *Crfr2* mRNA expression was not detected in the adBNST, the receptor may be expressed on presynaptic nerve terminals in the adBNST arising from other brain regions. Indeed there is evidence that CRF-R2-immunoreactive fibers in the BNST originate from the PVN (Dabrowska et al., 2011). This could explain the behavioral/physiological effects of CRF-R2 manipulation, despite a lack of mRNA expression.

The present findings together with the mpBNST data (Klampfl et al., 2014) demonstrate the tremendous heterogeneity of the BNST sub-regions in terms of behavior modulation by one neuropeptide family. Different behavioral outcomes of CRF-R manipulation in the ad- and mpBNST may be explained by different inputs and projection sites in the respective divisions (Dong et al., 2001a, Dong et al., 2001b, Dong and Swanson, 2004). Importantly, the anterior and posterior divisions of the BNST also vary in the distribution of CRF family members. CRF-ir cell bodies, fibres and nerve terminals are concentrated within the anterior BNST (Ju et al., 1989), whereas urocortin 3 (CRF-R2 ligand) is primarily found in the posterior part (Li et al., 2002). These different neuroanatomical/neurochemical profiles may underpin differential effects of CRF-R manipulation on maternal behavior depending on the site of action.

In conclusion, manipulation of the BNST CRF system exerted differential effects on maternal and anxiety-related behavior in an anterior-posterior fashion, independent of HPA axis activation. These results demonstrate the complexity of maternal neurophysiology and provide new insights into the potential causes of disturbed maternal behavior postpartum.

## Conflict of interest

The authors declare no competing financial interests.

## Contributors

All authors contributed equally to this work.

## Funding

This study was supported by the Deutsche Forschungsgemeinschaft (DFG BO 1958/8-1 to OJB). Work in PJB’s laboratory was supported by the BBSRC (BB/J004332/1).
