# Supplementary figures and images for: CRF-R1 activation in the anterior-dorsal BNST induces maternal neglect in lactating rats via an HPA axis-independent central mechanism
Source: Psychoneuroendocrinology. 2016 Feb;64:89–98. doi: 10.1016/j.psyneuen.2015.11.015 (PMC4712652; doi:10.1016/j.psyneuen.2015.11.015)

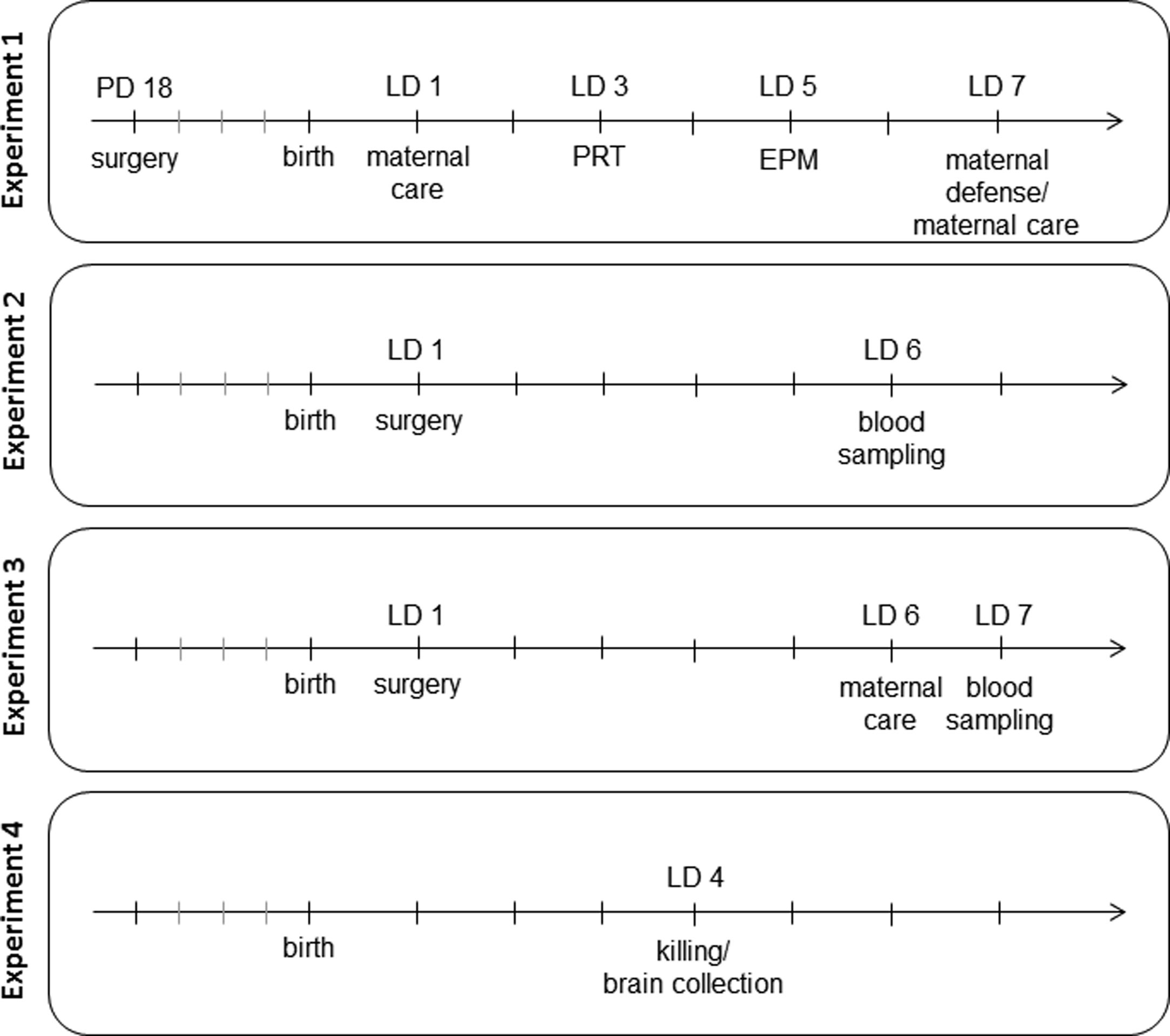

Supplement: Supplementary file 1 [file mmc1.jpg]
