# Supplementary material for: CRF-R1 activation in the anterior-dorsal BNST induces maternal neglect in lactating rats via an HPA axis-independent central mechanism
Source: Psychoneuroendocrinology. 2016 Feb;64:89–98. doi: 10.1016/j.psyneuen.2015.11.015 (PMC4712652; doi:10.1016/j.psyneuen.2015.11.015)
Supplement: Supplementary file 2 [file mmc2.docx]

SUPPLEMENTARY DATA

**Results**

**Experiment 1: Consequences of intra-adBNST manipulation of CRF-R1 or -R2 on other and non-maternal behaviors**

*Non-stress conditions on LD 1*

Non-maternal behaviors: Off-nest behavior differed significantly at t+30 min (Kruskal-Wallis H test; χ²(4) = 19.591, p<0.01), t+60 min (χ²(4) = 22.631, p<0.01), t+90 min (χ²(4) = 11.099, p=0.02) and t+300 min (χ²(4) = 21.187, p<0.01) between the different treatment groups. CRF-R1 agonist-treated dams displayed significantly more off-nest behavior at these time-points compared with VEH (p<0.01 for t+30 min, t+60 min and t+300 min, p=0.02 for t+90 min).

Locomotion differed significantly at t+30 min (Kruskal-Wallis H test; χ²(4) = 27.205, p<0.01), t+60 min (χ²(4) = 29.965, p<0.01), t+90 min (χ²(4) = 14.754, p<0.01) and t+300 min (χ²(4) = 25.018, p<0.01) between the different treatment groups. CRF-R1 agonist-treated dams displayed significantly more locomotion at these time-points compared with VEH (p<0.01 in each case).

Self-grooming differed significantly at t+30 min (Kruskal-Wallis H test; χ²(4) = 10.014, p=0.04), t+60 min (χ²(4) = 21.058, p<0.01) and t+90 min (χ²(4) = 10.066, p=0.03) between the different treatment groups. CRF-R1 agonist-treated dams displayed significantly more self-grooming at t+60 min compared to VEH (p<0.01).

Sleeping/resting differed significantly at t+30 min (Kruskal-Wallis H test; χ²(4) = 10.620, p=0.03), and t+90 min (χ²(4) = 10.620, p=0.03) between the different treatment groups; however, post hoc analysis revealed no further significant difference.

*Stress conditions on LD 7*

Non-maternal behaviors: No significant differences were detected for off-nest behavior, locomotion, self-grooming, and sleeping/resting (Table S2).

TABLES

Table S1. Effect of intra-adBNST CRF-R1 or -R2 specific agonist (ago) or antagonist (ant) treatment on non-maternal behaviors under non-stress conditions on LD 1. The occurrence of all off-nest behaviors was scored for 60 min before and 90 min after infusion (indicated by the dotted line) as well as during an additional 60 min period in the afternoon. Off-nest behavior is further divided into locomotion (including digging/burrowing and any explorative behavior in the home cage), self-grooming, and sleeping/resting. Dams received an acute bilateral infusion of either (i) vehicle (VEH), (ii) a CRF-R1 ago (CRF), (iii) a CRF-R1 ant (CP-154,526), (iv) a CRF-R2 ago (stresscopin), or (v) a CRF-R2 ant (astressin-2B) into the adBNST. Data is presented as mean ± SEM. n = 6 - 8 rats per group. ** p ≤ 0.01, * p ≤ 0.05 versus VEH (Kruskal-Wallis H test).

| Behavior | Group | Occurrence [n] | | | | | | | |
| --- | --- | --- | --- | --- | --- | --- | --- | --- | --- |
|  |  | -60 min | -30 min | +30 min | +60 min | | +90 min | +300 min | +330 min |
| Off-nest | VEH  CRF-R1 ago  CRF-R1 ant  CRF-R2 ago  CRF-R2 ant | 1.9±1.2  2.4±1.1  0.1±0.1  0.0±0.0  1.1±0.8 | 0.0±0.0  0.1±0.1  0.4±0.3  0.2±0.2  0.9±0.6 | 0.4±0.4  4.5±0.9**  2.9±1.2  4.8±2.9  2.4±0.9 | 0.4±0.4  4.9±1.0**  0.0±0.0  2.8±2.5  1.3±1.3 | 0.1±0.1  5.6±1.5*  0.6±0.4  3.0±2.4  0.6±0.4 | | 0.0±0.0  3.6±1.6**  0.3±0.3  0.0±0.0  0.0±0.0 | 0.3±0.2  1.0±0.5  0.9±0.4  1.3±1.0  1.3±0.9 |
| Locomotion | VEH  CRF-R1 ago  CRF-R1 ant  CRF-R2 ago  CRF-R2 ant | 0.9±0.4  0.8±0.5  0.1±0.1  0.0±0.0  0.0±0.0 | 0.0±0.0  0.0±0.0  0.4±0.3  0.2±0.2  0.0±0.0 | 0.3±0.3  2.9±0.6**  0.0±0.0  0.3±0.2  0.0±0.0 | 0.0±0.0  3.5±1.0**  0.0±0.0  0.2±0.2  0.1±0.1 | 0.0±0.0  2.8±0.8**  0.4±0.3  0.7±0.5  0.1±0.1 | | 0.0±0.0  2.3±1.0**  0.0±0.0  0.0±0.0  0.0±0.0 | 0.3±0.2  0.9±0.5  0.3±0.2  0.2±0.2  0.3±0.2 |
| Self-grooming | VEH  CRF-R1 ago  CRF-R1 ant  CRF-R2 ago  CRF-R2 ant | 0.0±0.0  0.1±0.1  0.0±0.0  0.0±0.0  0.3±0.3 | 0.0±0.0  0.0±0.0  0.0±0.0  0.0±0.0  0.0±0.0 | 0.1±0.1  1.1±0.4  0.0±0.0  0.8±0.7  0.3±0.3 | 0.0±0.0  2.9±1.0*  0.0±0.0  0.2±0.2  0.4±0.3 | 0.1±0.1  2.0±0.8  0.1±0.1  0.2±0.2  0.1±0.1 | | 0.0±0.0  0.1±0.1  0.0±0.0  0.0±0.0  0.0±0.0 | 0.0±0.0  0.1±0.1  0.1±0.1  0.0±0.0  0.1±0.1 |
| Sleeping/  resting | VEH  CRF-R1 ago  CRF-R1 ant  CRF-R2 ago  CRF-R2 ant | 0.0±0.0  1.4±1.0  0.0±0.0  0.0±0.0  0.0±0.0 | 0.0±0.0  0.0±0.0  0.0±0.0  0.0±0.0  0.0±0.0 | 0.0±0.0  0.0±0.0  0.0±0.0  3.2±2.0  0.0±0.0 | 0.0±0.0  0.0±0.0  0.0±0.0  2.5±2.5  0.0±0.0 | 0.0±0.0  0.0±0.0  0.0±0.0  1.3±1.1  0.0±0.0 | | 0.0±0.0  0.6±0.6  0.0±0.0  0.0±0.0  0.0±0.0 | 0.0±0.0  0.0±0.0  0.0±0.0  0.0±0.0  0.0±0.0 |

**Table S2. Effect of intra-adBNST CRF-R1 or -R2 specific agonist (ago) or antagonist (ant) treatment on non-maternal behaviors under stress conditions on LD 7.** The occurrence of all off-nest behaviors was scored for 60 min before and 60 min after the combined infusion with the maternal defense test (indicated by the dotted line). Off-nest behavior is further divided into locomotion (including digging/burrowing and any explorative behavior in the home cage), self-grooming, and sleeping/resting. Dams received an acute bilateral infusion of either (i) vehicle (VEH), (ii) a CRF-R1 ago (CRF), (iii) a CRF-R1 ant (CP-154,526), (iv) a CRF-R2 ago (stresscopin), or (v) a CRF-R2 ant (astressin-2B) into the adBNST. Data presented are group means ± SEM. n = 6 - 8 rats per group.

| Behavior | Group | Occurrence [n] | | | |
| --- | --- | --- | --- | --- | --- |
|  |  | -130 min | -100 min | 0 min | +30 min |
| Off-nest | VEH  CRF-R1 ago  CRF-R1 ant  CRF-R2 ago  CRF-R2 ant | 0.5±0.3  0.4±0.3  1.3±0.6  1.3±1.0  0.3±0.3 | 0.1±0.1  1.1±0.9  0.1±0.1  0.3±0.3  0.6±0.4 | 3.8±1.0  1.6±0.6  3.9±1.5  4.2±2.3  3.6±1.7 | 0.6±0.3  1.0±0.5  0.7±0.4  0.7±0.7  0.6±0.6 |
| Locomotion | VEH  CRF-R1 ago  CRF-R1 ant  CRF-R2 ago  CRF-R2 ant | 0.3±0.2  0.3±0.2  0.3±0.2  0.3±0.2  0.0±0.0 | 0.1±0.1  0.6±0.4  0.1±0.1  0.0±0.0  0.6±0.4 | 2.0±0.6  1.0±0.3  1.9±0.6  1.8±0.7  0.9±0.3 | 0.1±0.1  0.4±0.3  0.4±0.2  0.2±0.2  0.1±0.1 |
| Self-grooming | VEH  CRF-R1 ago  CRF-R1 ant  CRF-R2 ago  CRF-R2 ant | 0.3±0.2  0.1±0.1  0.1±0.1  0.0±0.0  0.0±0.0 | 0.0±0.0  0.1±0.1  0.0±0.0  0.2±0.2  0.0±0.0 | 0.5±0.3  0.3±0.3  0.7±0.3  0.8±0.5  2.1±1.3 | 0.1±0.1  0.1±0.1  0.1±0.1  0.2±0.2  0.3±0.3 |
| Sleeping/  resting | VEH  CRF-R1 ago  CRF-R1 ant  CRF-R2 ago  CRF-R2 ant | 0.0±0.0  0.0±0.0  0.0±0.0  1.0±1.0  0.3±0.3 | 0.0±0.0  0.1±0.1  0.0±0.0  0.0±0.0  0.0±0.0 | 0.9±0.6  0.0±0.0  0.6±0.6  0.2±0.2  0.4±0.2 | 0.3±0.2  0.0±0.0  0.0±0.0  0.3±0.3  0.0±0.0 |

**Table S3. Effect of i.v. ACTH infusion on maternal care on LD 6.** The occurrence of arched back nursing (ABN) and nursing was scored for 60 min before and 120 min after the infusion (indicated by the dotted line). Dams received an acute i.v. infusion of vehicle (VEH) or ACTH(1-39). Data presented are group means ± SEM. n = 7 - 10 rats per group.

| Behavior | Group | Occurrence [n] | | | | | | |
| --- | --- | --- | --- | --- | --- | --- | --- | --- |
|  |  | -60 min | -30 min | +0 min | +30 min | | +60 min | +90 min |
| ABN | VEH  ACTH | 8.9±1.1  8.5±1.6 | 6.0±2.3  9.0±1.6 | 8.1±1.7  10.5±1.3 | 7.7±2.3  10.5±1.4 | 9.1±1.9  8.5±1.5 | | 9.1±1.7  9.8±1.6 |
| nursing | VEH  ACTH | 1.3±1.0  10.7±1.4 | 10.0±1.2  10.1±1.4 | 11.7±1.2  12.4±1.0 | 11.6±1.9  13.8±0.4 | 12.9±0.9  12.7±0.8 | | 11.4±1.8  13.5±0.7 |

**Figures**

**Figure S1. Schematic overview of the experimental designs of experiments 1 – 4.** The time bars depict the different pregnancy (PD) or lactation days (LD) on which surgery, the behavioral or blood sampling experiments and the brain collection were performed. EPM, elevated plus maze; PRT, pup retrieval test.
